# Ashwagandha Root Extract Mitigates Fibromyalgia-like Symptoms via Neurochemical and Histological Modulation in Mice

**DOI:** 10.3390/cells14181478

**Published:** 2025-09-22

**Authors:** Razan Fawaz Hasanyn, Ashwaq H. Batawi, Mona A. AL-Thepyani, Reham Tash, Asma Almuhammadi, Ashwaq Hassan Alsabban, Badrah S. Alghamdi

**Affiliations:** 1 Department of Biological Science, Faculty of Science, King Abdulaziz University, P.O. Box 80200, Jeddah 21589, Saudi Arabia; rhasanyn@stu.kau.edu.sa (R.F.H.); amalmuhammadi@kau.edu.sa (A.A.); ahalsabban@kau.edu.sa (A.H.A.); 2 Neuroscience and Geroscience Research Unit, King Fahd Medical Research Center, King Abdulaziz University, Jeddah 21589, Saudi Arabia; mahalthepyani@kau.edu.sa (M.A.A.-T.); basalghamdi@kau.edu.sa (B.S.A.); 3 Department of Chemistry, College of Sciences & Arts, King Abdulaziz University, Rabigh 21911, Saudi Arabia; 4 Anatomy and Embryology Departement, Faculty of Medicine, King Abdulaziz University, Rabigh 25724, Saudi Arabia; rehamtash777@gmail.com; 5 Anatomy and Embryology Department, Faculty of Medicine, Ain Shams University, Abassia, Cairo 11566, Egypt; 6 Unit of Neurological Disorders, Princess Al-Jawhara Center of Excellence in Research of Hereditary Disorders (PACER.HD), Faculty of Medicine, King Abdulaziz University, Rabigh 25724, Saudi Arabia; 7 Clinical Physiology Department, Faculty of Medicine, King Abdulaziz University, Jeddah 21589, Saudi Arabia

**Keywords:** *Withania somnifera*, fibromyalgia, behavior symptoms, neurochemical markers, hisopathology

## Abstract

Fibromyalgia syndrome (FMS) is a chronic disorder marked by widespread musculoskeletal pain, fatigue, mood disturbances, and cognitive impairments. Current treatments primarily focus on symptom management. Ashwagandha (*Withania somnifera*), a traditional Ayurvedic herb, is known for its adaptogenic and neuroprotective properties. This study evaluated the protective effects of the methanolic root extract of Ashwagandha (ARE) in a reserpine-induced fibromyalgia model in male Swiss albino mice. Mice received oral ARE (100 mg/kg) for 17 days and reserpine (0.5 mg/kg, subcutaneously) for three consecutive days to induce fibromyalgia-like symptoms. Behavioral assessments included Von Frey, tail suspension, rotarod, and Y-maze tests. Histological analysis was conducted on the hippocampus and thalamus; however, neurochemical analysis focused on markers such as serotonin, norepinephrine, IL-1β, TNFα, MDA, and NO. Results indicated that ARE significantly reduced pain and depressive-like behavior and improved motor function (*p* < 0.0001); however, no significant changes were observed in open-field locomotion. Histological examination revealed protection of Ashwagandha against neurodegeneration and improved hippocampal integrity, accompanied by increased serotonin and norepinephrine levels and decreased pro-inflammatory cytokines. These findings suggest that Ashwagandha root extract may offer therapeutic benefits for managing fibromyalgia symptoms.

## 1. Introduction 

Fibromyalgia syndrome (FMS) is a chronic, multifactorial condition marked by widespread musculoskeletal pain, persistent fatigue, sleep disturbances, cognitive impairments, and psychological distress, all of which significantly reduce patients’ quality of life and lead to substantial functional disability [1]. According to the International Classification of Diseases 11th Revision (ICD-11), FMS is classified as chronic primary pain under the category of chronic widespread pain [2]. The global prevalence of FMS is estimated at 2–4%, with a notably higher incidence among women—particularly those aged 70 to 79 years, where prevalence reaches 7.4%—making it 8 to 9 times more common in females than in males [3].

FMS is widely regarded as a central sensitization disorder characterized by a dysfunction in neural circuits responsible for nociceptive processing. This includes altered perception, transmission, and modulation of pain signals, especially in the locomotor system [4]. Neurochemical imbalances associated with FMS include decreased levels of inhibitory neurotransmitters such as serotonin and norepinephrine, and elevated concentrations of excitatory substances like glutamate and substance P—these are found in cerebrospinal fluid at levels two to three times higher than normal [5]. These abnormalities contribute to the hallmark symptoms of fibromyalgia, such as generalized hyperalgesia and allodynia [6]. As reported by Alfaro-Rodríguez et al., disturbances in neurotransmitters are closely related to mood disorders and pain-related issues [7]. A deficiency in serotonin may increase pain sensitivity in fibromyalgia patients by impairing their ability to modulate pain effectively [8]. Additionally, Jurado-Priego et al. identified a correlation between elevated norepinephrine levels and poorer physical health status in individuals with fibromyalgia [6].

Cytokines are small signaling molecules secreted by various cell types that play a pivotal role in intercellular communication, particularly in immune responses and inflammatory processes [9]. In fibromyalgia (FM), serum levels of pro-inflammatory cytokines such as interleukin-6 (IL-6), interleukin-8 (IL-8), interleukin-1 beta (IL-1β), and tumor necrosis factor-alpha (TNF-α) are frequently elevated, whereas concentrations of anti-inflammatory cytokines are typically reduced [9]. Interestingly, Ernberg et al. reported significantly lower plasma IL-1β levels in FM patients compared to healthy controls, suggesting variability in cytokine profiles across patient subgroups [10]. In contrast, a more recent study by González-Álvarez et al. found that elevated IL-1β levels were associated with increased pain intensity and greater functional disability, highlighting the potential role of IL-1β in the modulation of FM symptom severity [11]. TNF-α, a key pro-inflammatory cytokine predominantly secreted by immune cells such as macrophages, has also been found at elevated levels in some FM patients, further supporting a possible link between systemic inflammation and the chronic pain characteristic of the condition.

Oxidative stress results from an imbalance between reactive oxygen species (ROS) production and the antioxidant defense system, leading to cellular damage and contributing to various diseases [12]. Biomarkers of oxidative stress include ROS-modified molecules and altered antioxidant components. Malondialdehyde (MDA), a lipid peroxidation product, is widely used to assess oxidative stress and has been linked to greater symptom severity in fibromyalgia (FM), including pain and fatigue [13]. Atamer et al. reported significantly higher MDA levels in FM patients compared to controls [14]. Nitric oxide (NO), involved in processes such as neurotransmission and inflammation, also shows altered levels in FM. While Atamer et al. observed decreased NO in FM patients, Andrabi et al. reported elevated NO levels, which correlated with higher FIQR (fibromyalgia impact questionnaire revised) scores [14,15]. Additionally, FM patients exhibited significantly lower antioxidant enzyme activities, further supporting the presence of an oxidative–antioxidative system imbalance in this population [15].

Given the absence of a definitive treatment for FMS, ongoing research has sought alternative approaches to alleviate its symptoms and improve patient outcomes. One such potential therapeutic agent is *Withania somnifera* (Ashwagandha), a traditional Ayurvedic herb known for its adaptogenic, anti-inflammatory, and neuroprotective properties [16]. The bioactive compounds in Ashwagandha, primarily withanolides, alkaloids, and sitoindosides, have demonstrated significant anti-stress, anti-anxiety, and antidepressant effects in both clinical and preclinical studies [17]. Despite these promising properties, no prior studies have explored the prophylactic potential of Ashwagandha in the context of fibromyalgia.

This study was designed to evaluate the potential role of *Withania somnifera* (L.) Dunal root extract to exert protective effects against fibromyalgia syndrome (FMS) symptoms by modulating neurotransmitter levels, reducing neuroinflammation and oxidative stress, and preserving brain tissue integrity. Therefore, the current work aimed to estimate protective effects in a male mouse model of fibromyalgia through behavioral, histological, and biochemical assessments following 17-day administration of the extract.

## 2. Materials and Methods

### 2.1. Animals

All animal procedures were approved by the Ethical Committee of King Abdulaziz University (Unit of Biochemical Ethics) and conducted in accordance with institutional and international guidelines (Ethical Approval Number: 384-24/ 5 December 2024). A total of 80 male Swiss mice (SWR/J strain), aged 9–10 weeks and weighing 22–27 g, were obtained from the Animal House Unit at King Fahad Medical Research Center. Mice were housed five per cage in polypropylene cages with paddy husk bedding, replaced daily. They were maintained under standard conditions (12 h light/dark cycle, controlled ambient temperature) with ad libitum access to standard rodent chow and water.

### 2.2. Methanolic Extraction of Ashwagandha Roots 

Ashwagandha root powder (300 g) was obtained from Organic India Co., Ltd. via the iHerb website ([https://cn.iherb.com/search?kw=Ashwagandha%20root%20powder], accessed on 17 January 2024) and was subjected to methanolic extraction. The powder was immersed in 1.5 L of methanol and homogenized using an Ultra-Turrax T50 IKA Labotechnik homogenizer (IKA, Wilmington, NC, USA; shaft G45ME) for 15 min at 6000 rpm, with 5 min pauses after every 3 min to prevent overheating. The homogenate was filtered through standard filter paper, and the filtrate was concentrated under reduced pressure at 40 °C using a rotary evaporator (Sigma-Aldrich, Fremont, CA, USA) until complete removal of methanol was achieved. So, no residual methanol was present in the administered extract to avoid any risk in the biochemical histological analyses. The extraction was repeated twice to ensure complete exhaustion of the plant material. The final yield was 22.46 g (8.62%) of a yellow-brown oily extract, which was confirmed to be solvent-free and stored at 4 °C until use.

### 2.3. Experimental Design

**Figure 1:**
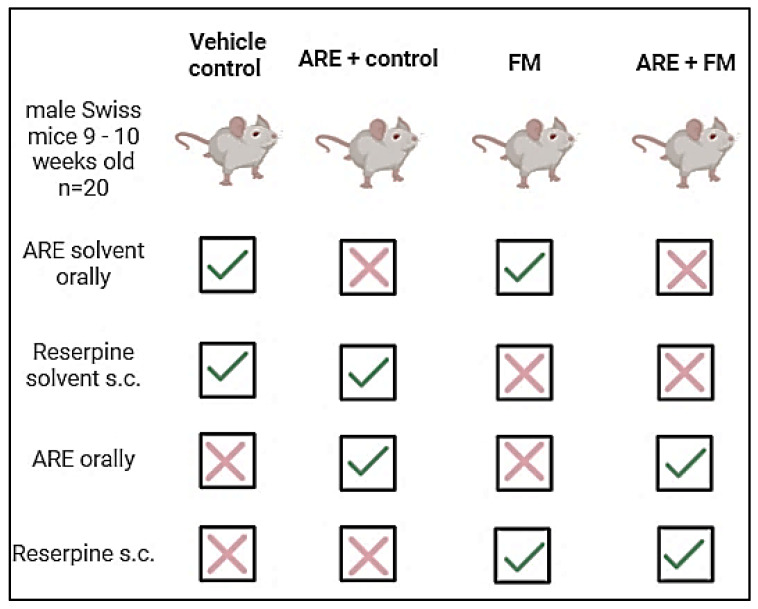
The group design in the experiment.

**Figure 2:**
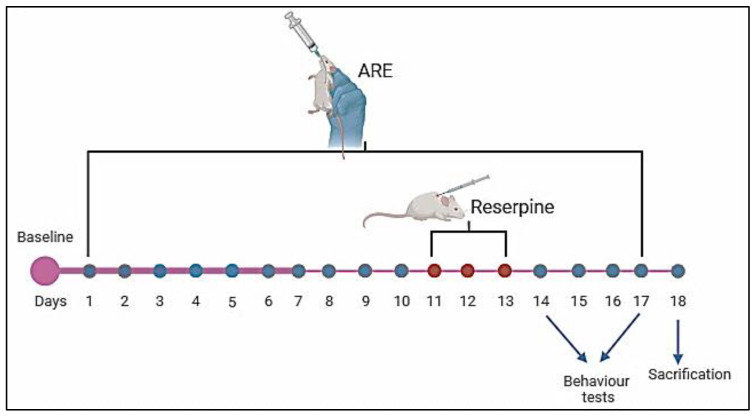
Timeline of the experiment.

Animals (*n* = 80) were randomly divided into four groups (*n* = 20 each). Treatments were given either orally (p.o.) or subcutaneously (s.c.) as follows: Group 1: Vehicle Control, Distilled water (vehicle for Ashwagandha) was administered orally once daily for 17 days, along with the vehicle of reserpine (Sigma-Aldrich, St. Louis, MO, USA) (0.5% glacial acetic acid diluted with distilled water) s.c. for three days (days 11–13). Group 2: Ashwagandha Extract (ARE), Ashwagandha root extract (100 mg/kg, p.o.) was given once daily for 17 days. From days 11–13, animals also received s.c. injections of 0.5% glacial acetic acid, 3 h after ARE administration [18]. Group 3: Fibromyalgia (FM): Distilled water (p.o.) was given once daily for 17 days, plus reserpine (0.50 mg/kg, s.c.) dissolved in glacial acetic acid once daily for three days (days 11–13) to induce fibromyalgia-like symptoms [19]. Group 4: Ashwagandha+Fibromyalgia (ARE + FM), Ashwagandha extract (100 mg/kg, p.o.) was administered for 17 days together with reserpine (0.50 mg/kg, s.c.) on days 11–13. All treatments were administered at the same time each day. Figure 1 and Figure 2 summarize the treatment schedule and timeline.

### 2.4. Body Weight Assessment

The body weight of each mouse was recorded daily between 07:00 and 09:00 a.m. throughout the experimental period. Changes in body weight were calculated as a percentage using the following formula:

$$\mathrm{Change}\;\mathrm{in}\;\mathrm{body}\;\mathrm{weight}\;(\%)\;=\;\frac{Day\;weight-initial\;weight}{initial\;weight}\times 100$$

The initial weight was defined as the baseline measurement obtained on Day 1 of the study, while the day-specific weight referred to the corresponding daily measurement.

### 2.5. Behavioral Assessments

#### 2.5.1. Pain Behavior Evaluation

##### Von Frey Test

Mechanical sensitivity was evaluated using the von Frey test as described by [20]. Mice were placed in mesh-bottom cages, and calibrated monofilaments were applied perpendicularly to the plantar surface of the hind paw. A positive response, such as licking, shaking, or paw withdrawal, was recorded. The up-down method was used to determine mechanical thresholds, starting with a 0.6 g filament. Responses were documented and analyzed using UpDown Reader software 9 (UDReader) [20].

##### Hot Plate Test

The hot plate test was employed to assess the antinociceptive effect of ARE. Mice were placed on a metal surface maintained at 55 ± 1 °C, and behavioral responses such as paw licking or jumping were recorded [21]. A cut-off time of 30 s was set to prevent tissue damage, and this was considered the maximum latency.

#### 2.5.2. Depression-like Behavior

##### Tail Suspension Test

The tail suspension test was used to assess depression-like behavior, as described by Can et al. [22]. Mice were suspended by the tail using adhesive tape attached to a horizontal wire, approximately 50 cm above the surface. Immobility time over a 6-min period was recorded through manual video analysis. Mice were considered immobile when showing no active movement. The test was conducted 60 min after oral administration of ARE.

##### Forced Swimming Test

The forced swimming test (FST) was used to evaluate depression-like behavior in mice. Mice were placed individually in a glass cylinder (10 cm diameter × 20 cm height) filled with water (23–25 °C) to a depth of 14 cm and allowed to swim for 6 min. Immobility time was recorded during the final 4 min. Immobility was defined as the absence of active escape behaviors [23].

##### Splash Test

The splash test was performed to assess self-care behavior. Mice were individually housed and sprayed on the dorsal coat with a 10% sucrose solution using a syringe. Grooming behavior was recorded for 5 min following application [24].

#### 2.5.3. Locomotor Activity

##### Open Field Test

Locomotor activity was assessed using the open field test. Each mouse was placed in the center of a 45 × 45 × 34 cm acrylic arena and allowed to explore freely for 3 min under low-intensity lighting in a sound-attenuated room. Velocity (cm/s) and total distance moved (TDM, cm) were recorded using the EthoVision XT8A system (Noldus, Wageningen, The Netherlands) [25].

##### Rotarod Test

The rotarod test was used to assess motor coordination and balance [26]. Mice were first trained to remain on the rod for 30 s at 9 rpm. One hour later, performance was evaluated by recording the latency to fall over a 300-s trial at a constant speed of 20 rpm [23].

##### Grip Strength Test

Grip strength was measured to assess forelimb muscle function. Mice were held by the tail and allowed to grasp a metal bar connected to a grip strength meter. A steady backward pull was applied, and the peak force was recorded. The average of three consecutive trials was used for analysis [27].

#### 2.5.4. Spatial Working Memory

##### Spontaneous Alternation Test

Spatial working memory was assessed using the Y-maze spontaneous alternation test [28]. The Y-maze consisted of three identical arms (A, B, and C) arranged in a Y-shape. Each mouse was placed at the center, and behavior was video-recorded from above. Mice were allowed to explore freely for 8 min. Spontaneous alternation was defined as consecutive entries into all three arms without repetition, indicating intact working memory, and was assessed using the following formula:

$$Spontaneous\;alternation=\frac{Total\;alternation}{Total\;entry-2}\times 100.$$

### 2.6. Sampling 

At the end of the experimental treatment, mice were anesthetized using isoflurane, and blood samples were collected via cardiac puncture. Subsequently, euthanasia was performed by cervical dislocation, and the brains were carefully dissected. Ten brain samples from each group were immediately fixed in 10% neutral-buffered formalin for 48 h, followed by standard histopathological processing. The remaining ten brain tissue samples were rapidly frozen and stored at −80 °C for subsequent biochemical analyses.

### 2.7. Histological Analysis

Following 48 h of fixation in 10% neutral-buffered formalin ((pH 7.0 ± 0.2), Sigma-Aldrich^®^, St. Louis, MO, USA, Catalog # HT501128-4L), brain samples were processed for histology. Tissues were dehydrated in ascending ethanol (Sigma-Aldrich, St. Louis, MO, USA) concentrations, cleared in xylene (Sigma-Aldrich, St. Louis, MO, USA), and embedded in paraffin (Sigma-Aldrich, St. Louis, MO, USA) [29]. Sections were stained with hematoxylin and eosin (H&E) (Sigma-Aldrich, St. Louis, MO, USA) and examined under a light microscope (Olympus, Tokyo, Japan), focusing on the hippocampus and thalamus.

### 2.8. Biochemical Analysis

Brain tissues were homogenized (1:10 *w*/*v*) in cold buffer (0.25 M sucrose, 1 mM EDTA (Sigma-Aldrich, St. Louis, MO, USA), 5 mM MOPS, 0.1% ethanol, pH 7.2), then centrifuged at 1000× *g* for 10 min at 4 °C. The resulting supernatants were used to assess neurotransmitters, proinflammatory cytokines, and oxidative stress markers [19].

#### 2.8.1. Serotonin and Norepinephrine Determination

Serotonin and norepinephrine levels were quantified in homogenized brain tissues using commercial ELISA kits (Solarbio, Beijing, China) according to the manufacturer’s instructions. Absorbance was measured at a wavelength of 450 nm using a microplate reader (Bio-Base, Qingdao, Shandong, China), and concentrations were determined from standard curves.

#### 2.8.2. Measurement of IL-1β and TNF-α Levels

Levels of IL-1β and TNF-α were measured in homogenized brain tissues using mouse-specific ELISA kits (Solarbio, Beijing, China). After incubation with capture antibodies and streptavidin-HRP, absorbance was recorded at a wavelength of 450 nm. Cytokine concentrations were calculated based on standard curves.

#### 2.8.3. Analysis of MDA and NO 

Malondialdehyde (MDA) and nitric oxide (NO) levels were assessed in homogenized brain tissues using ELISA kits (BT LAB, Ningbo, Zhejiang, China). After sample preparation and incubation with antibodies and enzyme conjugates, absorbance was read at 450 nm, and analyte levels were determined via calibration curves.

### 2.9. Statistical Analysis

Data were analyzed using GraphPad Prism v10 (GraphPad Software, San Diego, CA, USA). Group differences were evaluated by one-way or two-way ANOVA, followed by Tukey’s post hoc test. Results are expressed as mean ± SEM, with *p* < 0.05 considered statistically significant.

## 3. Results

### 3.1. Effect of Ashwagandha Root Extract (ARE) on Body Weight 

**Figure 3:**
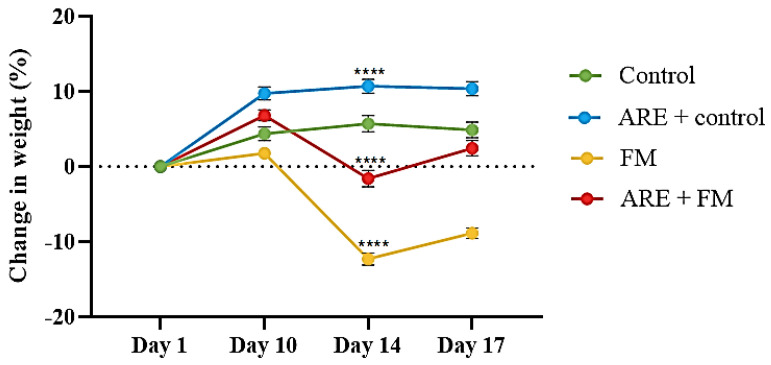
Changes in body weight of mice dosed with (100 mg/kg) of ARE for 17 days and injected subcutaneously with vehicle or reserpine (0.5 mg/kg) for 3 consecutive days. Data were expressed as the Mean ± SEM for all groups. * Indicates a significant change, where **** *p* < 0.0001. A two-way ANOVA measure was used, followed by Tukey’s post hoc test.

The results proved that administration of Ashwagandha root extract (ARE) at a dose of 100 mg/kg resulted in a significant increase (*p* < 0.0001) in body weight over the 17-day experimental period compared with control mice (Figure 3). In contrast, the Fibromyalgia (FM) group (reserpine-treated group) showed a significant reduction in body weight relative to the control group (*p* < 0.0001). Notably, co-administration of ARE with reserpine (ARE + FM group) resulted in a significant improvement in body weight compared to the FM group alone (*p* < 0.0001) (Figure 3).

### 3.2. Behavioral Study

#### 3.2.1. Mechanical Allodynia and Evoked Thermal Nociception in Mice

**Figure 4:**
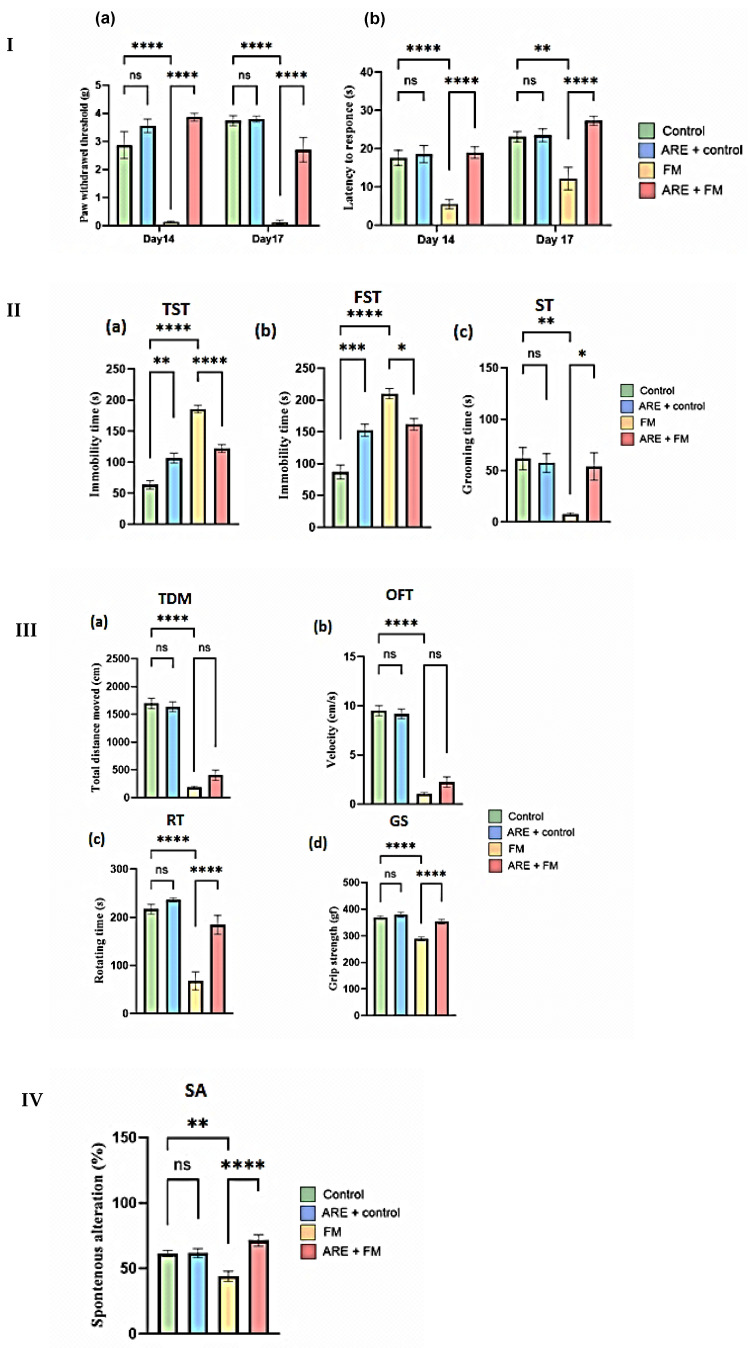
Behavioral analyses including (**I-a**) paw withdrawal threshold in the Von Frey test; (**I-b**) latency in response to hot stimulus on a hot plate test; (**II-a**) immobility time during tail suspension test (TST); (**II-b**) immobility time during forced swimming test (FST); (**II-c**) grooming time during splash test (ST); (**III-a**) total distance moved; (**III-b**) velocity in the open field test; (**III-c**) rotating time in the rotator test; (**III-d**) grip strength; and (**IV**) spontaneous alternation using a Y-maze. Data were expressed as the Mean ± SEM for all groups. ns: Not significant; * Indicates a significant change, where * *p* < 0.05, ** *p* = 0.0024, *** *p* < 0.001 and **** *p* < 0.0001. A two-way ANOVA measure was used, followed by Tukey’s post hoc test.

Mechanical allodynia was measured using manual von Frey filaments on days 14 and 17 following administration of reserpine or vehicle for three consecutive days (Figure 4I). The results revealed no significant difference in paw withdrawal thresholds between the ARE+control and control groups. However, the fibromyalgia (FM) group exhibited a significant reduction in withdrawal threshold compared to the control group (*p* < 0.0001). Notably, treatment with ARE significantly increased the paw withdrawal threshold in the ARE+FM group compared to the FM group (*p* < 0.0001).

To assess the effect of ARE on reserpine-induced thermal hypersensitivity, the hot plate test was conducted to measure latency to nociceptive response. No significant differences were observed between the control and ARE+control groups. In contrast, the FM group exhibited a significantly reduced response latency compared to the control group (*p* < 0.0001 and *p* = 0.0024 on days 14 and 17, respectively). However, ARE administration in the FM group significantly increased response latency on both days compared to the FM group alone, indicating attenuation of thermal hypersensitivity (Figure 4I).

#### 3.2.2. Antidepressant-like Effects of ARE

The potential antidepressant-like effects of Ashwagandha were assessed using the tail suspension test (TST), forced swimming test (FST), and splash test (ST). In the TST and FSTs, there was a significant increase in immobility time in the ARE+control group compared to the control group (*p* = 0.0036, *p* < 0.0001, respectively) (Figure 4(IIa,b)). Similarly, the FM group showed a marked increase in immobility time compared to controls (*p* < 0.0001), whereas ARE treatment significantly reduced immobility time in the ARE+FM group compared to the FM group (*p* < 0.0001, *p* = 0.0150, respectively) (Figure 4(IIa,b)). In the splash test, grooming time did not differ significantly between the ARE+control and control groups. However, the FM group exhibited a significant reduction in grooming time compared to controls (*p* = 0.0073), which was significantly improved by ARE treatment in the ARE+FM group (*p* = 0.0285) (Figure 4(IIc)).

#### 3.2.3. Effect of ARE on Motor Function

Motor performance was assessed using the open field, rotarod, and grip strength tests. In the open field test, total distance moved (TDM) and velocity were significantly reduced in the FM group compared to controls (*p* < 0.0001). No significant differences were observed between the ARE+control and control groups, or between the ARE+FM and FM groups (Figure 4(IIIa,b)). In the rotarod test, the FM group exhibited significantly reduced latency to fall relative to the control group (*p* < 0.0001), while ARE treatment significantly improved motor performance in the ARE+FM group (*p* < 0.0001). No significant difference was noted between the ARE+control and control groups (Figure 4(IIIc)). Grip strength was also significantly decreased in the FM group compared to controls (*p* < 0.0001) and significantly improved in the ARE+FM group (*p* < 0.0001). ARE alone had no effect on grip strength in healthy mice (Figure 4(IIId)).

#### 3.2.4. Effect of ARE on Spontaneous Alternation

The results revealed a significant reduction in alternation in the FM group compared to controls (*p* = 0.0036). ARE treatment restored performance, with the ARE+FM group showing significant improvement versus FM alone (*p* < 0.0001). No significant difference was observed between the ARE+control and control groups (Figure 4IV).

### 3.3. Biochemical Results

#### 3.3.1. Modulation of Serotonin and Norepinephrine Levels by ARE

**Figure 5:**
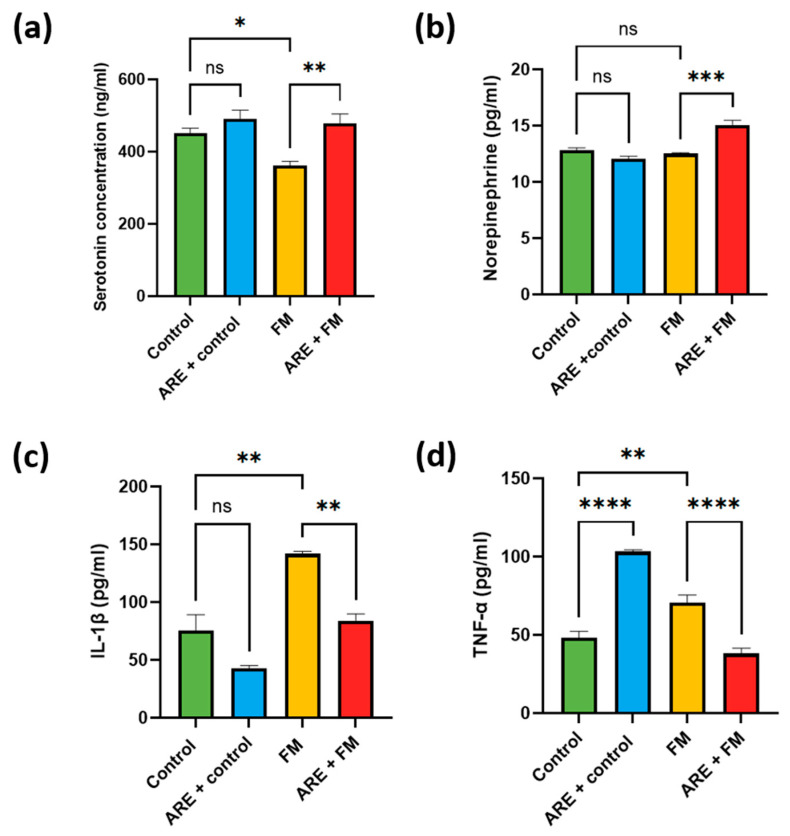
Effect of ARE on serotonin (**a**), norepinephrine (**b**), IL-1β (**c**), and TNF-α (**d**). Data were expressed as Mean ± SEM. ns: Not significant; * Indicates a significant change, where * *p* < 0.05, ** *p* < 0.01 and *** *p* < 0.001, **** *p* < 0.0001. One-way ANOVA measure was used, followed by Tukey’s post hoc test.

Brain analysis revealed that serotonin levels did not differ significantly between the ARE+control and control groups but were significantly reduced in the FM group (*p* = 0.0296). Treatment with ARE+FM significantly increased serotonin compared to the FM group (*p* = 0.0040) (Figure 5a). Norepinephrine levels showed no significant changes in ARE+control or FM groups versus control; however, the ARE+FM group exhibited a significant increase compared to the FM group (*p* = 0.0001) (Figure 5b).

#### 3.3.2. Alterations in Brain IL-1β and TNF-α Levels

IL-1β levels remained unchanged in the ARE+control group compared to the control but were significantly elevated in the FM group (*p* = 0.0013) and reduced in the ARE+FM group (*p* = 0.0045) (Figure 5c). TNF-α levels were significantly increased in both ARE+control (*p* < 0.0001) and FM groups (*p* = 0.0020) versus control, while ARE+FM treatment significantly reduced TNF-α levels (*p* < 0.0001) (Figure 5d).

#### 3.3.3. Alterations in MDA and NO Levels 

Malondialdehyde (MDA) and nitric oxide (NO) are critical biomarkers of oxidative stress in the brain. However, analysis revealed no statistically significant differences in the levels of MDA and NO among the experimental groups.

#### 3.3.4. Histological Alterations in the Hippocampus

**Figure 6:**
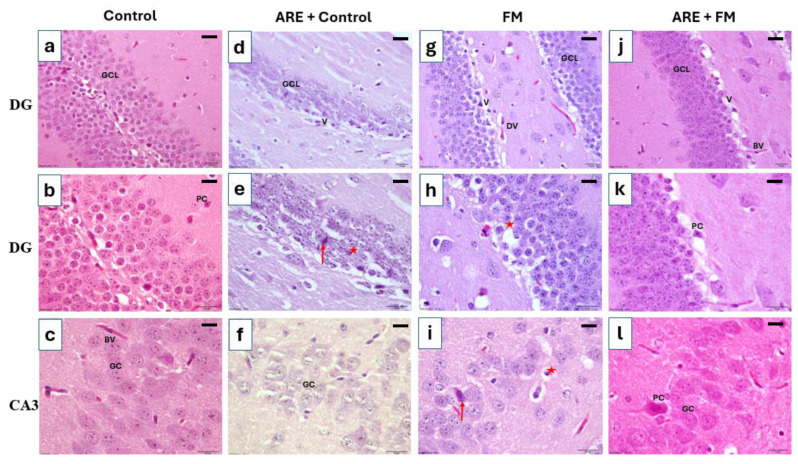
Photomicrographs of H&E-stained hippocampal sections illustrating histological changes across experimental groups. (**a**,**b**) Control group: Dentate gyrus (DG) shows a well-organized granular cell layer (GCL) composed of densely packed, rounded granule cells. Pyramidal cells (PC) exhibit large vesicular nuclei and pale basophilic cytoplasm. (**c**) CA3 region (control): Displays normal architecture with intact granular cells (GC). (**d**,**e**) ARE+control group: GCL shows disrupted architecture with variable-sized vacuoles (V), degenerated and shrunken granule cells (red star), and shrunken PCs with dark-stained cytoplasm (red arrow). (**f**) CA3 (ARE+control): Marked architectural loss and degeneration of GC. (**g**,**h**) FM group: GCL displays disorganization, prominent vacuolization (V), dilated blood vessels (DV), and degenerated GC (red star). (**i**) CA3 (FM group): Shows GC loss, open-faced nuclei (red star), and shrunken PCs with dark cytoplasm (red arrow). (**j**,**k**) ARE+FM group: GCL appears preserved with fewer vacuoles and normal blood vessels (BV). (**l**) CA3 (ARE+FM group): Exhibits normal morphology of both PC and GC, Scale bar= 100 µm.

Histological examination of the hippocampus revealed degenerative changes in the pyramidal and granular cell layers within the dentate gyrus and CA3 regions in both the ARE+control and FM groups compared to the control group. In contrast, the ARE+FM group exhibited reduced histopathological alterations compared to the FM group. Most pyramidal neurons retained normal morphology with vesicular nuclei, and the granular cell layer showed marked improvement following treatment with Ashwagandha root extract (Figure 6).

#### 3.3.5. Histological Alterations in the Thalamus

**Figure 7:**
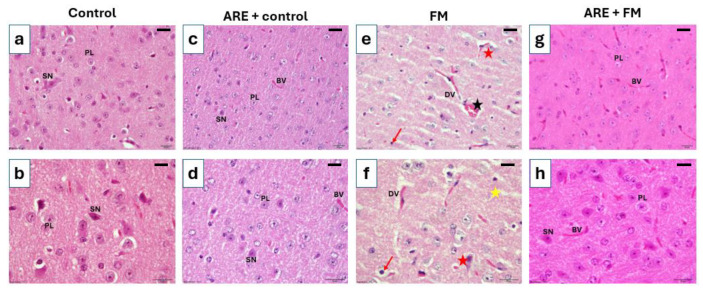
Photomicrographs of H&E-stained thalamic sections across experimental groups. (**a**,**b**) Control group: Normal thalamic architecture with densely packed principal cells (PL) exhibiting rounded nuclei, and intact sensory neurons (SN) with pyramidal-shaped cell bodies and prominent nuclei. (**c**,**d**) ARE+Control group: Normal appearance of PL and SN, along with intact blood vessels (BV). (**e**,**f**) FM group: Evident neuronal degeneration, including ruptured neurons (red star), pyknotic nuclei (red arrows), dilated blood vessels with hemorrhage (black star), and detached white matter fibers (yellow star). (**g**,**h**) ARE+FM group: Marked histological improvement with normal appearance of PL and SN, comparable to the control group, Scale bar = 100 µm.

Histological analysis of the thalamus revealed normal architecture in both the control and ARE+control groups, characterized by large principal cells with rounded nuclei, intact sensory neurons, well-organized white matter, and normal blood vessels. In contrast, the FM group exhibited marked degenerative changes, including pyknotic nuclei, disrupted white matter fibers, dilated blood vessels, and focal hemorrhage. Notably, the ARE+FM group showed substantial histological improvement, with thalamic features closely resembling those of the control group (Figure 7).

## 4. Discussion

Fibromyalgia is a chronic disorder of unclear etiology that has shown increasing prevalence over recent decades [30]. Despite substantial research, no definitive diagnostic test or universally effective treatment is currently available. Its complexity necessitates a multidisciplinary management approach, incorporating both pharmacological and non-pharmacological strategies [31]. Given the limitations and side effects of conventional therapies, such as tolerance and dependence associated with antidepressants and anticonvulsants, there is a growing interest in plant-based interventions with better safety profiles. Natural compounds have recently attracted attention for their potential as novel analgesic and antinociceptive agents in fibromyalgia management [32].

In the current study Fibromyalgia mice model was induced by reserpine (0.5 mg/kg/day, s.c.) in which it led to a significant decrease in body weight on days 14 and 17, consistent with prior findings of Park et al., who found a weight reduction in mice treated with reserpine (0.5 mg/kg i.p.) for 10 days [33]. Moreover, administration of reserpine for three consecutive days induced mechanical allodynia and thermal hyperalgesia, as evidenced by a reduced paw withdrawal threshold in the von Frey test and decreased latency in the hot plate test—findings consistent with previous FM models [34].

Although the reserpine-induced model has been widely used to mimic fibromyalgia-like features such as pain hypersensitivity, fatigue, and depressive-like behavior, it does not fully replicate the complex and multifactorial nature of human fibromyalgia, which involves genetic, hormonal, immune, and environmental contributions. The study is limited by the use of the reserpine-induced fibromyalgia model, which primarily reflects monoamine depletion and depressive-like symptoms, rather than the full spectrum of fibromyalgia pathophysiology.

One of the main objectives of this study was to evaluate the protective effect of *Withania somnifera* (ashwagandha) against reserpine-induced fibromyalgia in mice. Ashwagandha treatment counteracted this effect, supporting previous reports of its role in weight regulation [35]. They proved that administration of Ashwagandha led to significant improvements in body weight of adults at 4 and 8 weeks. Although Ashwagandha has not been previously studied specifically in FM models, our results indicate that its root extract significantly attenuated pain responses in both von Frey and hot plate tests, suggesting an antinociceptive effect. These findings align with earlier studies demonstrating Ashwagandha’s analgesic potential in other pain models [36].

Depression and anxiety are core non-pain symptoms of FM [9]. In line with previous reports, reserpine administration led to striatal dopamine depletion, resulting in depressive-like behaviors, including decreased locomotion and reduced exploratory activity [37]. In this study, Ashwagandha’s antidepressant-like effects were evaluated using the tail suspension test (TST), forced swim test (FST), and splash test (ST). Reserpine increased immobility in the TST and FST and reduced grooming behavior in the ST, consistent with earlier findings [38]. However, treatment with Ashwagandha significantly reversed these behavioral deficits, supporting its potential role in alleviating affective symptoms associated with FM.

In this work, we aimed to evaluate the antidepressant-like effects of methanolic *Withania somnifera* root extract using validated behavioral models relevant to FM-associated depression. Ashwagandha is a well-known medicinal plant with reported neuroprotective, immunomodulatory, antioxidant, and anti-inflammatory properties. Treatment with Ashwagandha significantly reduced immobility time in both the TST and FST, and significantly increased grooming behavior in the ST, indicating an improvement in depressive-like symptoms. These findings are consistent with previous reports highlighting the anti-inflammatory and antidepressant effects of Ashwagandha in standardized depression models [39].

Reserpine-treated mice exhibited characteristic FM-like symptoms, including general weakness, hypolocomotion, and reduced food intake, with some showing muscle twitching [40]. Behavioral assessments in the present study confirmed motor impairments, as evidenced by prolonged immobility in the open field, reduced grip strength, and shorter latency in the rotarod test, findings that align with earlier studies implicating reserpine in monoamine depletion and neuromuscular dysfunction [38]. In contrast, Ashwagandha treatment significantly improved motor performance and muscle strength in the FM model. These results are in agreement with previous studies demonstrating Ashwagandha’s role in enhancing physical performance, muscle mass, and reducing inflammatory markers such as CRP, IL-6, and TNF-α in both human and animal models [41,42]. The improvements observed in grip strength and motor coordination further support its potential in alleviating FM-related motor deficits. Interestingly, ARE improved rotarod and grip strength performance but did not influence open-field locomotion. This divergence may indicate that ARE enhances neuromuscular coordination and muscle strength rather than general exploratory activity. Alternatively, it may reflect a false positive due to multiple testing, which warrants cautious interpretation. Further studies using complementary behavioral assays will be needed to confirm the specificity of these effects.

The Y-maze test was employed in the current study to assess spatial working memory impairment following reserpine administration. Reserpine-treated mice exhibited a significant reduction in spontaneous alteration, indicative of cognitive deficits resembling those observed in FM. These impairments are likely associated with increased oxidative stress, as reserpine has been shown to elevate reactive oxygen species (ROS) in the brain through monoamine degradation, which impairs learning and memory functions [43].

Neurotransmitter imbalances, particularly involving serotonin and norepinephrine, are central to the altered pain perception and cognitive dysfunction in FM [44]. Consistent with its mechanism of action, reserpine significantly reduced brain serotonin levels in our model by inhibiting the vesicular monoamine transporter (VMAT), preventing vesicular storage, and promoting cytoplasmic degradation by monoamine oxidase (MAO) [45]. Ashwagandha root extract markedly increased serotonin levels, supporting previous findings that associate its antidepressant activity with serotonergic modulation [46].

While reserpine is known to deplete norepinephrine, dopamine, and serotonin by disrupting their vesicular storage, our results showed no significant change in norepinephrine levels between the FM and control groups [45]. This unexpected outcome may be attributed to compensatory mechanisms such as enhanced synthesis or release of norepinephrine, as well as the timing of sample collection, capturing transient fluctuations. Variability in dosage, treatment duration, or analytical methods may also explain the discrepancy [47].

Proinflammatory cytokines such as IL-1β and TNF-α are elevated in fibromyalgia (FM), contributing to neuroinflammation and central sensitization, which amplifies pain perception and is linked to fatigue and cognitive dysfunction [48]. In line with these findings, our study showed increased brain levels of IL-1β and TNF-α in the reserpine-treated group, indicating an activated neuroinflammatory state.

Treatment with Ashwagandha root extract significantly reduced IL-1β and TNF-α levels in the ARE+FM group, supporting the anti-inflammatory role of Ashwagandha in attenuating chronic pain-related neuroinflammation. Interestingly, elevated TNF-α levels were observed in the ARE+control group. This may be attributed to Ashwagandha’s known immunostimulatory properties, as previous studies have shown increased levels of cytokines such as IFN-γ, IL-2, and GM-CSF in normal mice treated with Ashwagandha extract, suggesting a possible context-dependent proinflammatory effect [49].

Oxidative stress is also implicated in FM pathogenesis, with elevated nitric oxide (NO) and malondialdehyde (MDA) levels reported in patients [14]. However, in the current study, no significant differences in NO or MDA levels were detected among the experimental groups. These unexpected findings may reflect the limitations of NO and MDA as reliable indicators of localized or acute oxidative stress. Their high variability and short-lived nature may reduce their sensitivity in detecting subtle or transient oxidative changes in FM models [50].

The hippocampus plays a central role in memory, cognition, mood regulation, stress response, and pain processing [51]. This study evaluated the protective effects of Ashwagandha root extract against reserpine-induced histopathological alterations in the brain. In the present study, we chose a dose of 100 mg/kg ARE guided by previous reports demonstrating neuroprotective and analgesic activity at this dose. However, the lack of dose–response assessment in the present study limits interpretation of dose–efficacy relationships. The histological changes observed in the ARE-only group may indicate that lower doses could be more appropriate for clinical translation. Further studies employing a range of doses are necessary to determine the minimum effective dose and to confirm the safety margin of ARE.

In this study, reserpine administration resulted in significant structural damage in the hippocampus, including degeneration of the dentate gyrus, disruption of the granular cell layer, vacuolation, ruptured pyramidal cells, and dilated blood vessels with reduced cellular density in the CA3 region. These findings align with previous studies linking reserpine to hippocampal neurodegeneration and with clinical evidence of reduced hippocampal volumes in FM patients, associated with cognitive deficits such as impaired memory, attention, and executive function [23,51]. In contrast, treatment with Ashwagandha significantly attenuated these histological abnormalities, preserving the integrity of the granular and pyramidal cell layers in the dentate gyrus and CA3 region. These neuroprotective effects are consistent with earlier studies demonstrating Ashwagandha’s role in preventing hippocampal neuronal loss via anti-inflammatory and antioxidant mechanisms [52].

Interestingly, mild structural changes were observed in the dentate gyrus of the ARE+control group. This unexpected outcome may be related to factors such as high dosage, prolonged exposure, or individual physiological differences. Excessive intake of certain phytochemicals has been reported to exert excitotoxic or oxidative stress-related effects in vulnerable brain regions, including the hippocampus [53]. In addition to hippocampal pathology, the thalamus, integral to motor coordination through its connections with the cerebellum, basal ganglia, and motor cortex, also showed damage in the FM group. Reserpine induced degeneration of primary thalamic neurons and loss of sensory neurons, consistent with previous findings in FM models [19]. Ashwagandha treatment ameliorated these changes, supporting its neuroprotective action, likely mediated through antioxidant and anti-inflammatory pathways [39].

## 5. Conclusions

This study demonstrates the potential protective effects of *Withania somnifera* (Ashwagandha) root extract against reserpine-induced fibromyalgia (FM) in mice. Ashwagandha significantly alleviated FM-related symptoms, including pain hypersensitivity, depressive-like behaviors, motor impairments, and cognitive deficits. These improvements were supported by behavioral, biochemical, and histopathological findings, suggesting its antinociceptive, antidepressant, anti-inflammatory, and neuroprotective properties. Given the limitations of current FM treatments, Ashwagandha may represent a promising natural therapeutic candidate for integrative FM management.

## Data Availability

Data supporting the findings of this study are available from the corresponding authors upon reasonable request.

## Abbreviations

FMS	Fibromyalgia syndrome
FIQR	Fibromyalgia impact questionnaire revised
ARE	Ashwagandha
IL-1β	Interleukin-1 beta
TNFα	Tumor necrosis factor alpha
MDA	Malondialdehyde
NO	Nitric oxide
CRP	C-reactive protein
ROS	Reactive oxygen species
TST	Tail suspension test
FST	Forced swim test
ST	Splash test
